# Methods for Detecting the Environmental Coccoid Form of *Helicobacter pylori*

**DOI:** 10.3389/fpubh.2015.00147

**Published:** 2015-05-28

**Authors:** Mahnaz Mazaheri Assadi, Parastoo Chamanrokh, Chris A. Whitehouse, Anwar Huq

**Affiliations:** ^1^Environmental Biotechnology Group, Biotechnology Department, Iranian Research Organization for Science and Technology, Tehran, Iran; ^2^Maryland Pathogen Research Institute, University of Maryland, College Park, MD, USA; ^3^The Geneva Foundation, Frederick, MD, USA

**Keywords:** *Helicobacter pylori*, environmental coccoid form, detection methods, LAMP, PCR

## Abstract

*Helicobacter pylori* is recognized as the most common pathogen to cause gastritis, peptic and duodenal ulcers, and gastric cancer. The organisms are found in two forms: (1) spiral-shaped bacillus and (2) coccoid. *H. pylori* coccoid form, generally found in the environment, is the transformed form of the normal spiral-shaped bacillus after exposed to water or adverse environmental conditions such as exposure to sub-inhibitory concentrations of antimicrobial agents. The putative infectious capability and the viability of *H. pylori* under environmental conditions are controversial. This disagreement is partially due to the fact of lack in detecting the coccoid form of *H. pylori* in the environment. Accurate and effective detection methods of *H. pylori* will lead to rapid treatment and disinfection, and less human health damages and reduction in health care costs. In this review, we provide a brief introduction to *H. pylori* environmental coccoid forms, their transmission, and detection methods. We further discuss the use of these detection methods including their accuracy and efficiency.

## Introduction

*Helicobacter pylori* is recognized as the most common cause of gastritis, peptic and duodenal ulcers, and gastric cancer (1, 2). For many years, the transmission dynamics of *H. pylori* largely remained unknown and has thus gained the interest of many researchers around the world. In many studies, contaminated water is implicated as a source of transmission of this pathogen that colonizes more than 50% of humans (3). Water supplies contaminated by sewage with bodily fluids or feces from infected people have been considered as a potential source of *H. pylori* infection (4, 5). The transmission of *H. pylori* may occur from person to person both via the oral-to-oral and fecal-to-oral routes (6). Some previous studies showed a positive correlation between *H. pylori* infection and consumption of untreated or low-quality drinking water suggesting the waterborne transmission of *H. pylori* (7–9). *H. pylori* transforms from the normal spiral-shaped bacillary form into the coccoid form when it is exposed to water in adverse conditions (5, 10). Like other Gram-negative bacteria, the coccoid forms of *H. pylori* are also usually in viable but non-culturable (VBNC), less virulent, and less likely to colonize and induce inflammation than the spiral forms. It has been demonstrated that bacteria in the VBNC state are able to maintain their metabolic activity and pathogenicity (11) as well as may revert to active re-growth conditions (12, 13). It is well known that the detection of *H. pylori* in coccoid forms is difficult using traditional methods (14). It was long assumed that the bacterial cells were dead when they were no longer able to form colonies on routine culture media. We now know this assumption is too simplistic and there are many situations where bacterial cells lose culturability but remain viable and are potentially able to regrow. Recently, investigators have demonstrated that the coccoid forms of *H. pylori* can be cultured on enrichment culture (15). Epidemiological studies suggest that the level of sanitation, particularly, water sanitation influences the probability of infection with *H. pylori*. The risk of *H. pylori* infection was suggested to be 2–13 times higher in people who drink untreated river or well water and swim in rivers, streams, or pools than those who drink municipal tap water and do not swim in such environments (8, 9, 16, 17).

*Helicobacter pylori* has been detected from drinking water (4, 18–20) as well as sea water (5). One *H. pylori* strain stored in deep ground water or in natural seawater at 4°C was observed to survive significantly longer than the same strain stored in nutrient-rich media (21). Several studies indicate that *H. pylori* may survive as culturable forms for weeks in water and may survive longer in natural systems than in artificial nutrient-rich systems (14, 22). Only a few studies reported the detection of *H. pylori* coccoid form in environmental water samples. In one study, the bacterium was found in a municipal wastewater canal on the U.S.–Mexico border, which was suggestive to be a fecal–oral route of contamination (4). In another study, *H. pylori* coccoid form was identified from a seawater sample (23). Furthermore, Samra et al. (24) examined 600 drinking water samples collected by water and sanitation agencies from ground-drilled water in different localities. In this review, we summarize the current approaches to detect the environmental coccoid form of *H. pylori* and discuss their sensitivity, specificity, and accuracy.

## VBNC State and Environmental *H. pylori* Coccoid Forms

Viable but non-culturable state is a bacterial response to some forms of natural adverse conditions such as nutrient starvation (25), extreme temperatures (26), incubation outside of the permissive pH or saltiness ranges for cell growth (27, 28), high- or low-osmotic concentrations (29), variable oxygen concentrations (30), exposure to food preservatives (31), and exposure to visible light and UV irradiation (32). Shahamat et al. demonstrated the entrance of *H. pylori* into the VBNC state for the first time during laboratory studies in which cells were observed to become non-culturable in freshwater microcosms (33). Cells in the VBNC state typically demonstrate very low levels of metabolic activity, but on resuscitation become culturable again (5, 34–36). Many suggest that *H. pylori* persists in the environment in a VBNC form (21, 34, 37, 38) and there is only scattered evidence for reversion to the actively dividing form (39, 40). *H. pylori* is mostly found in a spiral shape within the human host, but it converts into a coccoid shape when is exposed to unfavorable environments (41). It has been suggested that based on evidence gathered over the last few years, the VBNC cells of human pathogens should be viewed as a potential hazard to public health rather than considered as dead cells (42). In addition, pathogens in a VBNC state may remain virulent or produce enterotoxins (43). An issue of much significance is to detect VBNC and viable-culturable (VC) cells by novel and more efficient methods. There is an urgent need for a method, which lowers selectivity, reduces bias from sample storage and incubation, and decreases assay time (44).

## Culture

**Figure 1:**
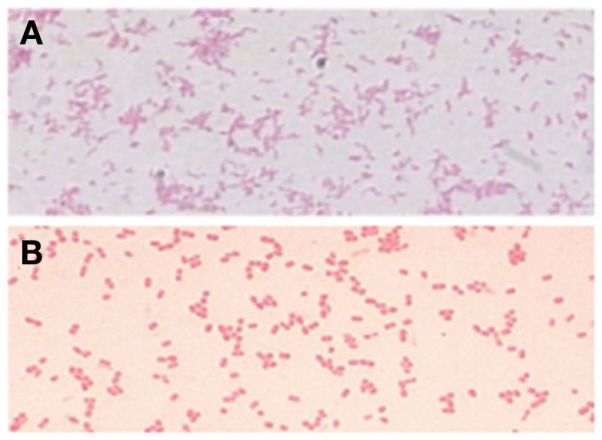
**Conversion from the *H. pylori* bacillary form (A) to the coccoid form (B) (15)**.

There are no established culture methods for the detection of *H. pylori* in the drinking water supplies (45). Despite efforts to produce a culture-specific, media-culturing *H. pylori* from drinking water has not been successful (46, 47). A simple plating medium was suggested to detect *H. pylori* in the environment (48). Several studies have reported that *H. pylori* enters into the coccoid form when exposed to a nutrient deficient environment (49), drug supplementation (50), pH change (39), abnormal temperature (51), or prolonged culture (52). It is believed that the spiral form (Figure 1A) is transformed immediately and rapidly into the coccoid form (Figure 1B) (34). The first successful isolation of *H. pylori* from environmental water using the enriched culture was from a municipal wastewater canal heavily contaminated with untreated raw sewage at the U.S.–Mexico border where *H. pylori* infection was reported frequently (4). However, the history of unsuccessful attempts to culture *H. pylori* from environmental waters led investigators to explore the use of molecular methods to detect and identify this organism.

## Autoradiography

Autoradiography was optimized and employed to detect metabolic activity of VBNC cells of *H. pylori* in water. Tritium-labeled cells of *H. pylori* showed the aggregations of silver grains associated with uptake by *H. pylori* of radiolabeled substrate. Temperature is a significant environmental factor for the viability of the organism in water. Autoradiography revealed that *H. pylori* remain viable at 4°C for 26 months. However, sterile water does not reflect the natural environment in which competition with naturally occurring populations of microorganisms can occur. Findings based on an autoradiography approach provided evidence supporting the hypothesis that there is a waterborne route of infection for *H. pylori* (51).

## Electron Microscopy

**Figure 2:**
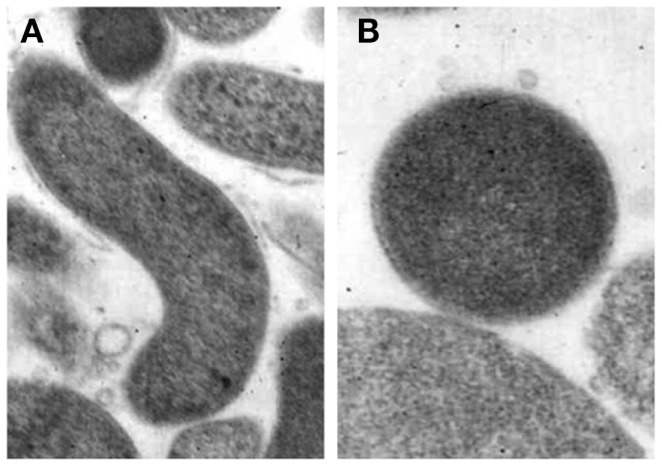
**Morphological appearances of *Helicobacter pylori***. **(A)** Rod-shaped **(B)** Coccoid form. Original magnification × 20,000 (58).

Coccoid forms have been divided into two types, a and b, by electron microscopy although the function of the two different coccoid forms of *H. pylori* is unclear. One possibility is that coccoid form in general represents a degenerating state of the organism (53). Kusters et al. (54) indicated that the coccoid cells of *H. pylori* were the morphological manifestation of bacterial cell death, observing the transformation process by electron microscopy. However, others suggested this form to be VBNC (55). Benaissa et al. (56) asserted that coccoid *H. pylori* was devoid of degenerative change. Willén et al. (57) studied morphologic conversion of *H. pylori* from spiral (Figure 2A) to coccoid (Figure 2B) form where scanning electron microscopy and transmission electron microscopy were employed.

## Fluorescent *In Situ* Hybridization

Fluorescent *in situ* hybridization (FISH) with ribosomal RNA oligonucleotide probes has been used successfully for the detection and identification of VBNC forms of bacteria (59). FISH was validated as a quick and sensitive method for the detection of *H. pylori* in environmental samples (60). In the U.S., actively respiring *H. pylori* from surface and well water has been detected using fluorescent antibody-tetrazolium reduction (FACTC) microscopy (18) and confirmed using species-specific polymerase chain reaction (PCR) (61). These findings helped to determine the presence of *H. pylori* in the natural environment and a possible waterborne route of transmission. Use of FISH provides an alternative to PCR detection of *H. pylori* in water (60) and raw bovine milk (62). These findings imply that at some point in time helicobacters have entered the water source but it is not possible for PCR or hybridization methods to establish if viable organisms are present although coccoids in VBNC forms may be transmitted via water (63, 64).

## DNA-Based Techniques

Among the molecular methods, PCR has been widely used for the diagnosis of *H. pylori* infection as well as the analysis of diversity, virulence, persistence, and resistance patterns of these bacteria (65) including detection of the organisms in environmental samples (66, 67). Specific target genes are selected to avoid cross-reactivity between *H. pylori* and other bacteria. For example, PCR targeting the *16S rRNA* gene, random chromosome sequences, the 26-kDa *species specific antigen* gene, the urease A (*ureA*) gene, and the urease C (*ureC*) gene or *glmM* gene have been used (68–70). Among these gene targets, PCR-based detection of the *ureC* gene appears to be the most promising for the detection of *H. pylori* (69).

The presence of *H. pylori* in drinking water, which was detected by PCR, has been reported from many different countries (71, 72). Despite the requirement for a microaerobic atmosphere, helicobacters can possibly survive for short periods in water in a VBNC coccoid form (40, 49), which would allow them to be transmitted via the water distribution system while remain undetectable by culture techniques. Moreover, recent findings suggest that *H. pylori* cells may be able to tolerate the levels of disinfectant normally used in water purification plants. Results from one study showed the presence of *H. pylori* from U.S. surface water (18). *H. pylori* DNA has also been amplified from drinking water samples in Japan (73), Mexico (74), and Peru (75), untreated well water in the U.S. (76), from water samples taken from a water delivery truck and two lakes near Repulse Bay in the Canadian arctic (77), and from drinking water storage pots in Gambia (70).

Clearly, PCR is the only way to demonstrate the presence of *H. pylori* in water supplies and seawater (5, 78). *H. pylori* could not be cultured and the cell membrane was disintegrated but nucleic acid was still detected by PCR (47, 61). Furthermore, because of its high sensitivity, PCR was suggested to be an appropriate method to detect organisms when they are in low numbers, slow growing, or non-culturable form (79).

Despite the findings of much research to identify *H. pylori* in water, it is important to consider the fact that the use of PCR and other molecular methods for the detection of pathogens in environmental samples suffers from a number of limitations. The most serious limitation is that PCR does not enable us to distinguish between live and dead cells. It also suffers from the fact that it is biased and time consuming (44).

Nayak and Rose (80) demonstrated that quantitative PCR (qPCR) could determine *H. pylori* concentrations in water. In this study, qPCR was shown to be a specific, sensitive, and rapid method to quantify *H. pylori* in sewage. Another study showed that coccoid forms, regardless of viability, are readily detected in small numbers by qPCR assays (81). Nayak and Rose (80) investigated the detection of *H. pylori* in sewage and water using a new qPCR method with SYBR green. Janzon et al. (81) detected *H. pylori* DNA in drinking and environmental water in Dhaka, Bangladesh, using highly sensitive real-time PCR. Sen et al. (82) developed an internal control for evaluation and standardization of a qPCR assay for the detection of *H. pylori* in drinking water. There are also reports of the failure to identify *H. pylori* in drinking water in the U.S. (83) and in drinking water or reclaimed wastewater in low-endemic developed countries such as Belgium, Spain, and Italy (84).

The *H. pylori* qPCR test has several advantages. First, it does not rely on culturing. Second, many samples can be analyzed quickly, since real-time qPCR instruments are easily available and it can analyze up to 384 samples in 2 h. Third, real-time qPCR removes many of the sources of human error from the analysis process and lessens the potential of contamination. The results of McDaniels et al. (83) support the idea that a rapid real-time qPCR may be useful for the screening of large numbers of drinking water samples for the presence of *H. pylori* at low concentrations. Due to metabolic and morphological changes that can prevent *H. pylori* cells in water from growing on conventional media, an *H. pylori*-specific TaqMan qPCR assay that uses a 6-carboxyfluorescein-labeled probe has been developed (83).

In addition, there are a number of studies reporting traces of *H. pylori* in various water sources, mainly using PCR-based methods, although the successful isolation of live *H. pylori* from river water (4) or marine zooplankton (5) has been reported. However, some of these studies were performed with river water, lake water, or seawater (20, 85, 86) rather than drinking water, and some used nested PCR (20, 78, 87), which may increase detection sensitivity but is more prone to contamination.

## Flow Cytometry

Flow cytometry is an analytical technique, which has the potential to make a distinction among the four physiological states of bacteria: reproductively viable, metabolically active, intact, and permeabilized. It can determine the proportions of VBNC and VC states and dead cells, based on membrane integrity of Gram-negative bacteria (44). The application of this technique makes rapid *in situ* analysis of single cells possible. In addition, using this technique along with staining techniques such as live/dead staining, it is possible to obtain qualitative data (88). Although the use of flow cytometry has revealed four physiological states, rapid approaches to distinguish between VBNC and VC cells are not yet available (44).

## Loop-Mediated Isothermal Amplification Method

Loop-mediated isothermal amplification (LAMP) is a promising technique that can overcome some of the technical shortcomings of PCR. LAMP is a novel gene amplification strategy in which all reactions are conducted under isothermal conditions (i.e., no need for thermocycling) using a single type of enzyme. This method has high-amplification efficiency and provides faster amplification times than PCR (65, 89). LAMP amplifies targeted DNA producing magnesium pyrophosphate as a by-product DNA amplification can be detected by turbidity measured via photometry due to the increase of magnesium pyrophosphate in solution (65, 90) or by SYBR green addition, which can change the color detectable with naked eyes without the need for expensive equipment. Also, the detection of DNA amplification can use manganese loaded calcein, which starts fluorescing upon mixing with manganese by pyrophosphate during *in vitro* DNA synthesis (91).

**Figure 3:**
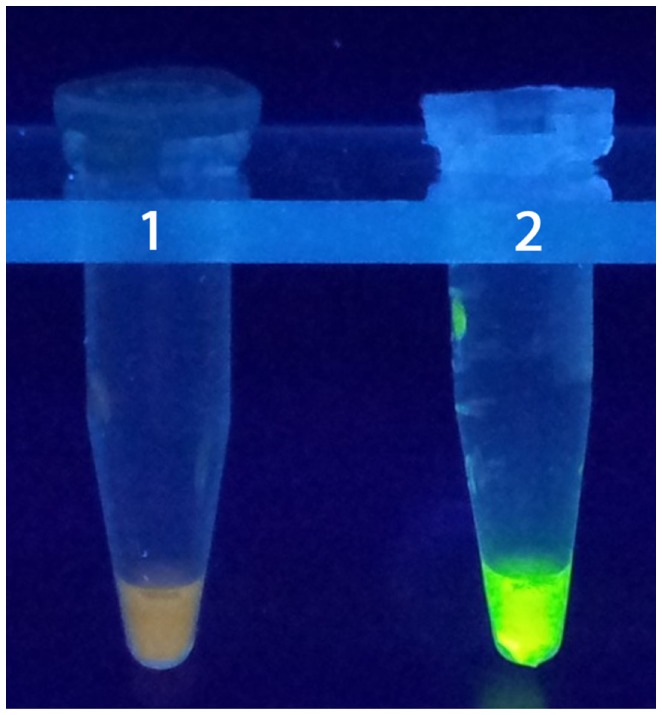
**LAMP test**. 1: negative control, 2: positive control (15).

It is, therefore, possible to detect the amplification of the products without gel electrophoresis using the white precipitate of magnesium pyrophosphate in the reaction mixture. This can be achieved due to high specificity and amplification efficiency of LAMP (65, 89). The simple operation of the LAMP assay offers advantages over currently available DNA probe and PCR methods (65). Although PCR methods are rapid and accurate compared to other detection techniques to detect coccoid forms of *H. pylori*, LAMP was found to be even more efficient detecting *H. pylori* coccoid forms in water samples (Figure 3) in terms of accuracy, rapidity, and sensitivity based on laboratory microcosm experiments (15), similar level of *H. pylori* detection was archived in the stomach biopsy samples, employing LAMP method (92).

## Next-Generation DNA Sequencing and Metagenomics

Metagenomics is the application of modern genomics techniques for studying microbial community directly in their natural environments (93). Importantly, metagenomics bypasses the need for laboratory cultivation of individual bacterial species, thereby allowing for the study of unculturable microorganisms (94). The presence of unculturable bacteria in the environment has been known for more than a century and has often been referred to as the “Great Plate Count Anomaly” (95). This concept is the condition in which there is a discrepancy – often by several orders of magnitude – between the sizes of a bacterial population estimated by culture compared to that observed under microscope. It may be fair to mention that the demonstration that *H. pylori* causing gastric ulcers and cancer helped to draw attention on the importance of the unculturable microbial world. Although spiral bacteria were observed in the gastric mucosa of dogs in 1893 and in humans in 1906 (96), and correlations between the occurrence of the bacteria and peptic ulcers were noted in 1938 (97), it was not until *H. pylori* was cultured when its role as the disease causing agent was accepted (98, 99).

By the mid-1980s, microbiologists began describing the phylogenetic diversity of microorganisms in “exotic” environments, such as oceans, deep sea vents, hot springs, soil, and others using molecular methods alone. Much of these culture-independent methods were based on isolating total DNA from an environmental sample, cloning the DNA into a suitable vector, transforming the clones into a host bacterium (i.e., producing a clone library), and screening the clones for a phylogenetic marker (e.g., 16S rRNA). Clones were then sequenced and 16S rRNA gene sequences cataloged to reveal the diverse taxa present in the sample (94). This technique has been used widely to identify bacteria and archaea from a variety of environments (100–103). Today, high-throughput, next-generation DNA sequencing has made this process vastly more efficient. Advancements in next-generation sequencing (and reduced costs) now provide a technical means by which to not only monitor environmental microbial communities but also to study the occurrence of pathogens in the natural environmental, especially those that are no longer culturable, such as VBNC forms of bacterial pathogens. Metagenomics coupled to next-generation sequencing has been used to study microbial communities in many natural environments, including coastal areas of Thailand (104), waters of the Puget Sound in the U.S. (105), freshwater and marine sediments along the Pearl River in China (106), among others. While none of these studies have specifically focused on non-culturable bacteria or the coccoid form of *H. pylori*, Zheng et al. developed methods for metagenomic analysis of *H. pylori* from old formalin-fixed and paraffin-embedded gastrointestinal biopsies using Roche 454 high-throughput pyrosequencing (107). It is reasonable to suggest that these approaches can be used for the detection and characterization of *H. pylori* in the natural aquatic environment.

## Conclusion

*Helicobacter pylori* is a significant human pathogen that is estimated to infect the gastric mucosa of half of the world’s population (108). The transmission dynamics of *H. pylori* are poorly understood; however, epidemiological data and the detection of *H. pylori* in a wide variety of natural aquatic environments points to waterborne transmission. Although, most attempts to culture *H. pylori* from water samples have proved unsuccessful, likely due to the presence of VBNC coccoid form, great tools to detect *H. pylori* in water samples, most commonly, PCR and qPCR are now available. As a powerful and accurate detection method for *H. pylori*, the qPCR technique provides diagnostic microbiology laboratories with a capacity to quantify and achieve a high degree of sensitivity and specificity of targets as compared to standard PCR. Other promising techniques for the detection of environmental *H. pylori* include the LAMP assay, which can be performed without a thermocycler and results can be visualized by eye (Figure 3). Perhaps one of the most exciting areas in microbiology, in the past decade, is the increasing use of next-generation DNA sequencing and metagenomics. As the instruments for DNA sequencing become more widespread and conveniently portable while the cost of sequencing continues to decrease, metagenomics will likely become the mainstream technology used in environmental microbiology and microbial ecology, including research into the transmission dynamics and potential reservoirs of environmental *H. pylori*. This awareness eventually will help public health official to take necessary action to protect people from *H. pylori* infection.

## Conflict of Interest Statement

The authors declare that the research was conducted in the absence of any commercial or financial relationships that could be construed as a potential conflict of interest.
